# Inflammatory modulation by cord blood stem cells prevented digit deformation in recessive dystrophic epidermolysis bullosa

**DOI:** 10.1016/j.ymthe.2025.08.038

**Published:** 2025-08-28

**Authors:** Morgan Anderson-Crannage, Alexander Nyström, Rahim Hirani, Edo Schaefer, Bruno Hochberg, Rebecca Kann, Jian Pan, Meijuan Tian, Hongwen Zhu, Wen Luo, Janet Ayello, Mitchell S. Cairo, Yanling Liao

**Affiliations:** 1 Department of Pediatrics, New York Medical College, Valhalla, NY 10595, USA; 2 Department of Cell Biology and Anatomy, New York Medical College, Valhalla, NY 10595, USA; 3 Department of Dermatology, University of Freiburg, Freiburg, Germany; 4 Freiburg Institute for Advanced Studies (FRIAS), University of Freiburg, Freiburg, Germany; 5 Department of Medicine, New York Medical College, Valhalla, NY 10595, USA; 6 Department of Pathology, Microbiology and Immunology, New York Medical College, Valhalla, NY 10595, USA

**Keywords:** unrestricted somatic stem cells, RDEB, inflammation, immunomodulation, IL-1, LIF

## Abstract

Recessive dystrophic epidermolysis bullosa (RDEB) is a hereditary dermal blistering disorder caused by mutations in the *COL7A1* gene encoding type VII collagen (C7), which progressively results in poor wound healing, fibrosis, and pseudosyndactyly. Using a C7 hypomorphic mouse model of RDEB, we demonstrated that inflammation critically drives disease progression and identified potential mechanisms by which human cord blood derived unrestricted somatic stem cells (USSCs) exert therapeutic benefit. Systemic USSC administration significantly mitigated early paw edema and prevented digit disfigurement; such effects were associated with promotion of wound healing macrophages. USSCs also deposited C7 at the dermal-epidermal junction, significantly promoted survival, and improved locomotor activity. Importantly, USSC treatment modulated relative balance between interleukin (IL)-1α and IL-1 receptor antagonist (IL-1Ra), resulting in significantly reduced IL1α/IL1Ra ratios and attenuated NK-кB signaling. Mechanistically, in response to inflammatory cues, USSCs secreted multiple paracrine factors, including leukemia inhibitory factor (LIF), granulocyte-colony-stimulating factor, and prostaglandin E2 (PGE2). Among these, LIF emerged as a key immunomodulator, simultaneously suppressing IL-1α and enhancing IL-1Ra expression. These findings highlight a novel mechanism in how LIF modulates IL-1-driven inflammation and suggest the potential therapeutic benefit of using USSCs to treat patients with RDEB.

## Graphical abstract

**Figure:**
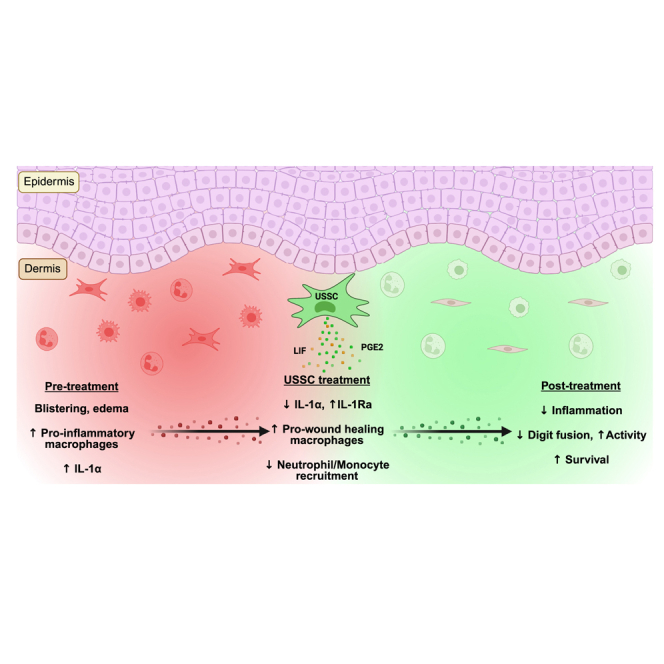

## Teaser

Unrestricted somatic stem cells (USSCs) ameliorated fibrosis, enhanced motility, and improved survival in an RDEB mouse model by modulating IL-1-driven inflammation and promoting wound-healing macrophages. LIF secretion from USSCs suppressed IL-1α and increased IL-1Ra in macrophages, highlighting the therapeutic potential of stem cell-mediated paracrine signaling in RDEB.

## Introduction

Epidermolysis bullosa encompasses a heterogeneous group of inherited dermal blistering disorders characterized by compromised integrity of the dermal-epidermal junction (DEJ).^1^ Among these, recessive dystrophic epidermolysis bullosa (RDEB) is one of the most severe forms, caused by mutations in the *COL7A1* gene, which encodes type VII collagen (C7).^2^ Patients with RDEB experience recurrent blistering of the skin, oral mucosa, gastrointestinal tract, and genitourinary tract. The cumulative effects of repeated blistering and lesions result in progressive and disfiguring scarring, leading to esophageal strictures and debilitating deformities, including pseudosyndactyly (mitten deformity).^1^ A life-threatening complication of RDEB is the development of aggressive cutaneous squamous cell carcinoma (cSCC), typically arising in early adulthood from chronic wounds or fibrotic scars.^3^ Treatment for RDEB typically revolves around managing symptoms, preventing infection, and minimizing complications. Interventions include non-adhesive dressings to protect the skin and surgery to release fibrous adhesions to address pseudosyndactyly.^4^

Multiple therapeutic approaches have been designed to achieve localized and systemic C7 replacement, including variations of cell, gene, and protein therapies. However, despite the emergence of potentially curative therapeutic approaches,^5^^,^^6^ the need to address secondary disease manifestations is becoming increasingly evident. Chronic inflammation, driven by repetitive dermal blistering and wounding, correlates with the rapid progression of RDEB to a severe, multiorgan fibrotic disease.^7^ Changes in the tissue microenvironment and systemic inflammatory milieu may impede the response to curative therapies. Thus, targeting chronic inflammation may not only alleviate RDEB symptoms but also improve the efficacy of C7 replacement therapies.^7^^,^^8^^,^^9^

Two mouse models of RDEB, C7 knockout (C7^KO^) and C7 hypomorphic (C7^hypo^), have proven to be invaluable in elucidating the inflammatory cascades involved in the condition. Time-course profiling of cytokines, utilizing both mouse models, unveiled multiple inflammatory cascades, presumably orchestrated by IL-1α.^10^ Additionally, C7^hypo^ mice with IL1 receptor (IL1R1) KO exhibited improved survival and delayed pseudosyndactyly, further implicating the involvement of IL-1 in RDEB pathology while also highlighting its potential as a therapeutic target.^11^ Supporting this notion, recent clinical investigations utilizing diacerein—a compound with IL-1β-inhibiting properties—improved wound healing in a 4-year-old RDEB patient.^12^

Unrestricted somatic stem cells (USSCs) are a rare, non-hematopoietic population in umbilical cord blood. They are not directly identifiable from cord blood but can be isolated based on their characteristic outgrowth as adherent colonies when cord blood mononuclear cells are cultured in the presence of 30% fetal bovine serum (FBS) and dexamethasone. USSCs are distinct from mesenchymal stem cells (MSCs) by several defining features, including expression of Delta-like homolog (DLK-1), which is associated with lack of adipogenic differentiation and greater proliferative capacity. Furthermore, USSCs lack HOX gene expression, exhibit a more plastic epigenetic state at pluripotency-associated gene loci, and possess broader differentiation potential.^13^^,^^14^^,^^15^^,^^16^^,^^17^^,^^18^^,^^19^^,^^20^ Importantly, USSCs secrete a variety of paracrine factors, such as leukemia inhibitory factor (LIF) and granulocyte-colony-stimulating factor (G-CSF) upon cytokine stimulation, which may promote regeneration and wound healing by modulating immune responses, angiogenesis, and matrix remodeling.^21^^,^^22^ USSCs have demonstrated therapeutic effects in various preclinical disease models, including intraventricular hemorrhage, spinal cord injury, myocardial infarction, liver cirrhosis, and lung injuries.^23^^,^^24^^,^^25^^,^^26^^,^^27^^,^^28^^,^^29^^,^^30^

USSCs also represent a promising therapeutic for RDEB. Previous studies from our group showed that systemic USSC administration improved median lifespan, enhanced dermal-epidermal adherence, and led to C7 deposition at the DEJ in C7^KO^ mice.^13^^,^^14^^,^^31^ The persistence of USSCs was short-term (less than 3 weeks) and did not elicit anti-C7 antibody production in recipient C7^KO^ mice. The early demise of these mice prevented a full assessment of the effects of USSCs on fibrotic progression and hindered investigation of the mechanisms underlying fibrosis development. In this study, we utilized C7^hypo^ mice, which are postnatally viable and recapitulate the disease progression of RDEB observed in human patients.^8^^,^^32^ Using this mouse model, we aimed to investigate the role of inflammation in fibrotic progression and to elucidate the effects and mechanisms of USSCs in modulating immune responses and suppressing fibrosis in RDEB.

## Results

### Severe inflammation and rapid paw deformation were prevented in C7^hypo^ mice by acute USSC treatment

**Figure 1:**
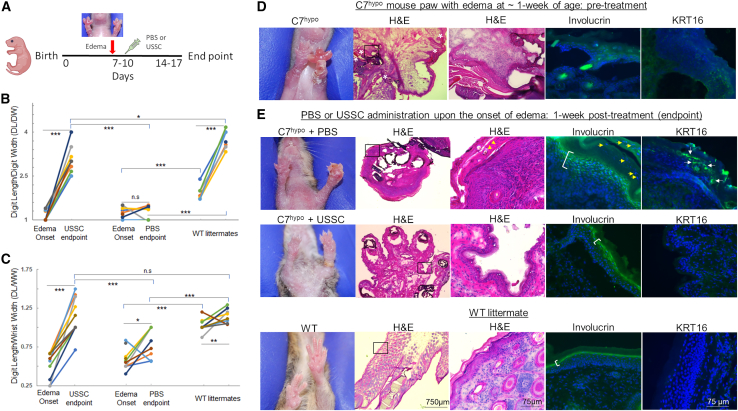
USSC administration prevented accelerated progression from severe inflammation to mutilating fibrosis in C7^hypo^ mice (A) Experimental timeline showing acute treatment of C7^hypo^ mice with USSCs. (B) Digit length/digit width (DL/DW) ratios and (C) digit length/wrist width (DL/WW) ratios in C7^hypo^ forepaws measured at baseline and 1 week following administration of PBS or USSCs, alongside age-matched wild-type (WT) controls (*n* ≥ 10 per group). (D and E) Representative hematoxylin and eosin (H&E) and immunofluorescence (IF) staining for involucrin (green), keratin 16 (KRT16; green), and nuclei (blue) in C7^hypo^ paw digits with edema (pre-treatment), 1 week post-PBS and 1 week post-USSC administration, compared with WT control. White asterisks (first panels) indicate hyperkeratosis; yellow arrows (second panels) denote retained nuclei in the stratum corneum. Areas with positive involucrin staining were indicated by brackets and KRT16-positive cells in post-PBS C7^hypo^ paw skin are shown by white arrows. Scale bars, 750 μm (4× magnification, upper panels) and 75 μm (20× magnification, lower panels). Statistical significance was determined by two-tailed unpaired Student’s t test. ∗*p* < 0.05, ∗∗*p* < 0.01, ∗∗∗*p* < 0.001; n.s., not significant. See also Figure S1 for additional characterization of inflammatory phenotypes in C7^hypo^ paws and identification of human USSCs by bioluminescent imaging, and Figure S2 for Masson’s trichrome staining.

Progressive mitten deformity, characterized by the gradual shortening and fusion of digits after age 1 month, commonly develops in C7^hypo^ mice, although disease severity varies.^9^^,^^32^^,^^33^ In our studies, a subset of C7^hypo^ mice exhibited edema, primarily affecting the front limbs from the digits to forearms, around 1 week of age (Figure 1A). Severe swelling led to an enlarged digit diameter and was associated with a significantly reduced digit length/digit width ratio (DL/DW) compared with wild-type (WT) littermates (Figure 1B). Additionally, the digit length/wrist width ratio (DL/WW) was also significantly lower than in WT (Figure 1C), due to wrist swelling. Histological hematoxylin and eosin (H&E) staining of the paws at the onset of edema revealed dermal-epidermal separation and fluid accumulation in the affected digits, along with hyperkeratosis (granular parakeratosis; shown by white asterisks) in digital folds and constricted regions connecting digits and forearms (Figure 1D). When left untreated or injected with phosphate-buffered saline (PBS) alone, these mice rapidly developed severe paw mutilation within a week (Figure 1E, top panels), with some cases progressing to necrosis and paw loss (Figure S1A). Further histological analysis of these paws revealed a densely packed extracellular matrix, inflammatory cell accumulation, and thickening of the stratum corneum with occasional nuclei retention (Figure 1E, top panels, yellow arrowheads in H&E-stained image). Moreover, multiple layers of involucrin-positive cells, indicative of differentiating cells in the stratum corneum, were noted and nucleated cells were still present at the final uppermost layer where desquamation occurs (shown by yellow arrows). In addition, KRT16, a marker associated with keratinocyte hyperproliferation (e.g., psoriasis), was detected in the epidermis of untreated C7^hypo^ paw skin. These results suggest that accelerated keratinocyte turnover and immature differentiation in the deformed C7^hypo^ paws are related to their transition from severe swelling to mitten deformity.

We first aimed to evaluate the efficacy of a single-dose USSC treatment in C7^hypo^ mice presenting with severe RDEB symptoms, using PBS injections as a control (Figure 1A). We monitored individual paws with edema and divided the C7^hypo^ mice with initial DL/DW ≤ 1.5 into PBS (1.25 ± 0.19) and 1 × 10^6^ USSCs (1.15 ± 0.18; *p* = 0.13) intraperitoneal administration groups. At the endpoint (1 week after administration), both DL/DW (3.01 ± 0.46) and DL/WW (1.15 ± 0.24) significantly increased in the USSC-treated mice, compared with the values at onset and the endpoint of the PBS injection group (Figures 1B and 1C). Migration of USSCs to paws was confirmed by bioluminescent imaging (Figure S1B). H&E staining of USSC-treated mouse paw skin revealed a less-dense dermal extracellular matrix (Figure 1E, mid panels). Additionally, the epidermal layer was thinner, lacked KRT16 expression, and exhibited similar involucrin staining to WT paw skin. We also performed Masson’s trichrome staining to assess collagen organization. In the PBS-treated C7^hypo^ mice after edema formation, the dermal matrix exhibited, in line with previous description of the fibrotic matrix in C7^hypo^ mice,^8^ a dense, highly aligned parallel collagen fiber pattern characteristic of excessive collagen deposition and fibrosis (Figure S2). In contrast, the dermis of USSC-treated C7^hypo^ mice showed a more loosely arranged, multidirectional collagen patterns, resembling that of a normal dermis.

Intriguingly, the distribution and level of mouse C7 did not significantly differ between mild and severe phenotypes of C7^hypo^ paws (Figure S3A). Moreover, pSmad2/3-mediated TGF-β signaling was not activated in C7^hypo^ paws with edema and there was no apparent difference in pSmad2/3 levels between paws that rapidly developed mutilating deformities and those with progressive fibrosis (Figures S3B and S3C). These findings suggest that other mechanisms play a more significant role in the development of digit deformities resulting from severe edema.

### USSC treatment promoted wound-healing macrophages in C7^hypo^ mice with edema

**Figure 2:**
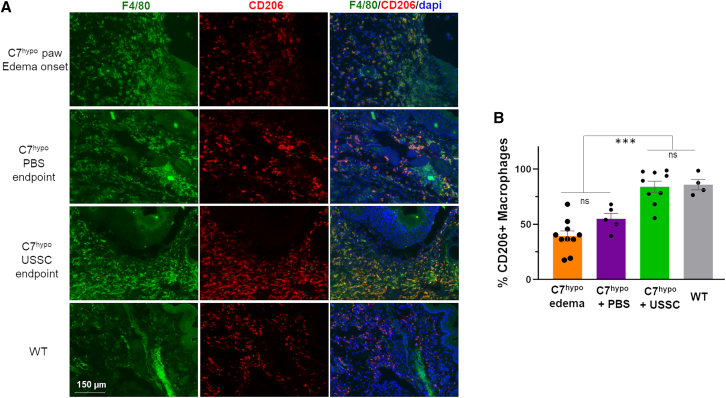
Modulation of macrophage phenotypes in C7^hypo^ mice with severe inflammation following USSC administration (A) Representative IF staining of paw skin from C7^hypo^ mice with severe inflammation (pre-treatment), 1 week following intraperitoneal injection of PBS or USSCs, and from 2-week-old WT controls. Staining includes F4/80 (pan macrophage marker; green), CD206 (M2 macrophage marker; red), and DAPI (nuclei; blue). Scale bar, 150 μm. (B) Quantification of CD206^+^ cells as a percentage of total F4/80^+^ macrophages in WT paw skin, swollen C7^hypo^ paws, fibrotic C7^hypo^ paws, and USSC-treated C7^hypo^ paws. Macrophage quantification was performed in a blinded manner, based on ≥4 randomly selected fields per mouse, with ≥4 mice per group. Statistical significance was determined using two-tailed unpaired Student’s t test. ∗*p* < 0.05, ∗∗*p* < 0.01, ∗∗∗*p* < 0.001; n.s., not significant. See also Figure S3 for additional C7 and pSmad2/3 immunohistochemistry analyses.

Macrophages have been proven to be a major immune cell involved in regulating inflammation and wound healing. Differentiation of macrophages to either a pro-inflammatory phenotype or a pro-wound-healing phenotype significantly influences how injured tissue heals.^34^ Therefore, we hypothesized that the resolution of edema and prevention of digit deformation in C7^hypo^ mice treated with USSCs were associated with changes in macrophage differentiation. Immunofluorescence (IF) analysis demonstrated F4/80^+^ macrophages were elevated in C7^hypo^ paws at edema onset when compared with WT paws (Figure 2A). This elevation in F4/80^+^ macrophages persisted among both PBS- and USSC-treated C7^hypo^ mice. However, USSC-treated C7^hypo^ mice exhibited a significantly higher percentage of CD206^+^ alternatively activated or wound-healing-like macrophages (84.03% ± 5.05%) than in pretreated (39.32% ± 4.66%) (*p* < 0.001) or PBS-treated mice (54.95% ± 5.02%) (*p* < 0.001), akin to WT (Figures 2A and 2B). These results suggested that the edema and deformation prevention facilitated by USSC treatment are related to their immunomodulatory activities promoting macrophages to express a pro-wound-healing phenotype. Macrophage polarization to a pro-wound-healing phenotype likely contributed to the improved extracellular matrix organization observed in USSC-treated C7^hypo^ mice.

### Acute USSC treatment accelerated excisional wound healing in C7^hypo^ mice

**Figure 3:**
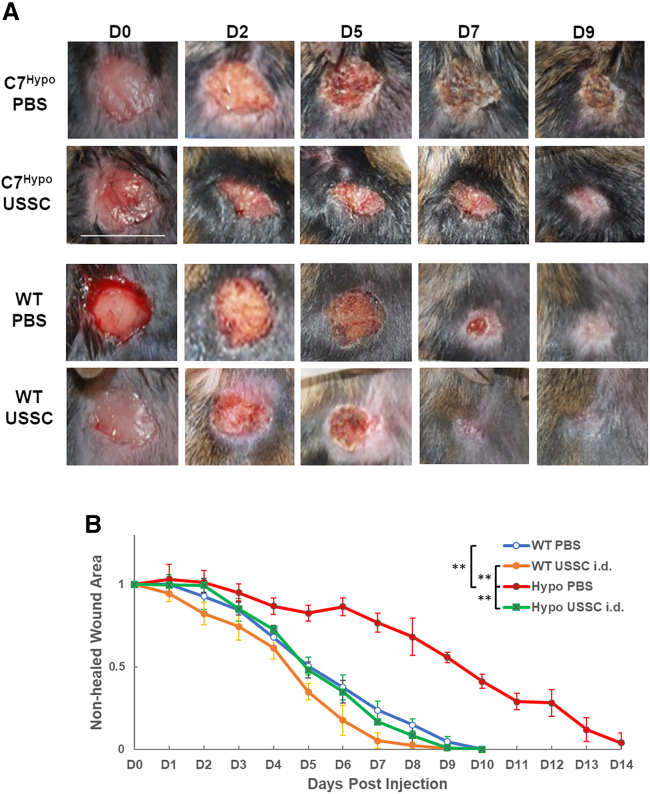
Acute USSC treatment accelerated excisional wound healing in C7^hypo^ mice (A) Representative images of dorsal wounds in C7^hypo^ mice treated intradermally (i.d.) with either PBS (C7^hypo^ PBS; *n* = 9) or unrestricted somatic stem cells (C7^hypo^ USSCs; *n* = 7), alongside WT mice treated i.d. with PBS (WT PBS; *n* = 12) or USSCs (WT USSC; *n* = 10). Scale bar, 1 cm. (B) Quantification of wound area over time, expressed relative to initial wound size, in WT and C7^hypo^ mice i.d. treated with either PBS or USSCs. Statistical significance between groups (WT PBS, WT USSCs, C7^hypo^ PBS, C7^hypo^ USSCs) was determined using Tukey’s multiple comparisons test. ∗∗*p* < 0.01. See also Figure S4 for the dynamics of WT wound healing after either i.d. or intravenous (i.v.) administration of USSCs and migration of USSCs to the wounds monitored by bioluminescent imaging.

Building upon our previous study, which demonstrated the ability of USSCs to facilitate wound healing in immunocompromised mice,^17^ we investigated the effects of USSC administration on the healing of acute wounds in C7^hypo^ mice. Consistent with previous reports,^35^^,^^36^^,^^37^ we demonstrated that wound healing in C7^hypo^ mice was significantly delayed compared with WT controls, but was significantly accelerated following a single dose of intradermally (i.d.) administered USSCs 24 h after wounding (*p* < 0.01) (Figures 3A and 3B). Indeed, USSC-treated C7^hypo^ wounds fully healed an average of 5 days earlier when compared with PBS-treated controls, resulting in wound-healing dynamics indistinguishable from WT controls. Notably, the effect of USSC treatment predominantly manifested at later stages, commencing 5 days after injection (Figures 3A and 3B). In WT mice with excisional wounds, both i.d. and intravenous (i.v.) delivery of USSCs significantly accelerated wound closure compared with PBS controls (Figure S4). The wound-healing kinetics were nearly identical between the two routes, suggesting that USSCs exert their reparative effects irrespective of the delivery method. Bioluminescent imaging demonstrated comparable localization of USSCs to the wound bed 24 h post-injection for both i.v. and i.d. routes. Residual bioluminescent signals were still detectable at the wound site at day 6 in both groups, indicating persistence of USSCs during the active phase of healing. Overall, these results support the use of acute USSC treatment in resolving impaired wound healing and edema in RDEB.

### Weekly USSC administration confers survival and locomotor benefits and modulated progressive inflammatory responses in C7^hypo^ mice

**Figure 4:**
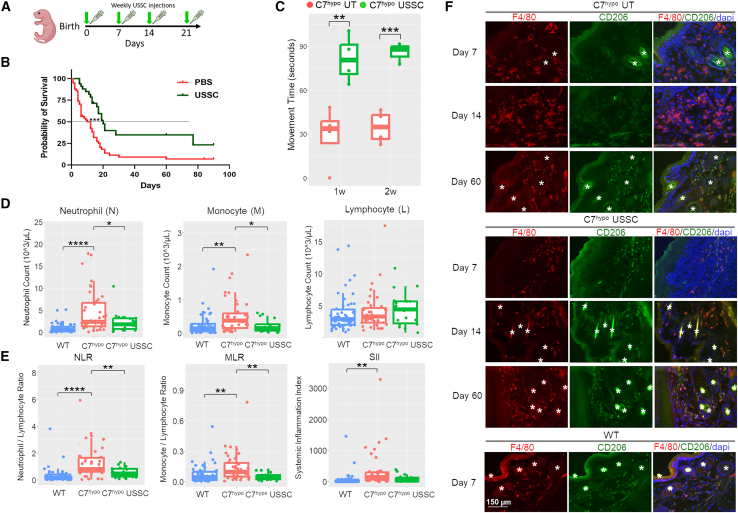
Weekly USSC administration confers survival and locomotor benefits, and reduces systemic inflammation in C7^hypo^ mice, associated with M2 macrophage polarization (A) Experimental timeline showing weekly intraperitoneal injection of USSCs in C7^hypo^ mice. (B) Kaplan-Meier survival analysis comparing median lifespans of C7^hypo^ mice treated with USSCs (20 days; *n* = 33, green line) or PBS (12 days; *n* = 57, red line). Statistical significance was determined using log rank (Mantel-Cox) test. (C) Movement was recorded over 5 min in untreated and USSC-treated C7^hypo^ mice at age 1 and 2 weeks (*n* = 3–4 per group). Movement time of mice for each recording was assessed in a blinded manner. Statistical significance was assessed using two-tailed paired t tests. See Video S1 for a representative example of movement analysis. (D and E) Total neutrophil, monocyte, and lymphocyte counts, along with neutrophil/lymphocyte ratio (NLR), monocyte/lymphocyte ratio (MLR), and systemic immune-inflammation index (SII) in WT mice (*n* = 50), C7^hypo^ mice (*n* = 39), and USSC-treated C7^hypo^ mice (*n* = 18). Statistical comparisons were performed using one-way ANOVA with Tukey’s post hoc correction. See also Figure S6 for NLR, MLR, and SII in all three experimental groups stratified by age. (F) Representative IF of F4/80 (red) and CD206 (green) in paw skin of C7^hypo^ mice at days 7, 14, and 60 following weekly PBS or USSC treatment, and from day 60 WT controls. White asterisks denote autofluorescence from hair. Nuclei were counterstained with DAPI (blue). Scale bar, 150 μm. ∗*p* < 0.05, ∗∗*p* < 0.01, ∗∗∗*p* < 0.001, ∗∗∗∗*p* < 0.0001. See also Figure S8 for CD163 expression in PBS or USSC-treated C7^hypo^ paw skin.

Based on the results from acute USSC treatments, we proceeded with weekly systemic USSC administration in C7^hypo^ mice (Figure 4A) to determine cumulative therapeutic benefits. We determined the median lifespan of C7^hypo^ mice under standard animal husbandry and care to be 12 days, consistent with previous findings.^33^ Weekly systemic administrations of USSCs in C7^hypo^ mice within 48 h of birth significantly elongated their median lifespan to 20 days (*p* < 0.001) (Figure 4B), consistent with its effects on the survival of C7 KO mice.^14^

While survival may no longer be a primary clinical concern in RDEB due to advances in care, it remains a meaningful indicator of overall health status in preclinical neonatal models, which exhibit severe disease burden. Thus, the observed survival benefit in USSC-treated mice suggests a broad improvement in systemic health. To further support this, we assessed the functional impact of USSC treatment using a behavior (locomotor activity) test, with a rationale that spontaneous movement reflects general health, energy levels, and pain sensitivity. USSC-treated C7^hypo^ mice exhibited significantly greater movement over a 5-min period than untreated controls at both 1 and 2 weeks of age (Figure 4C). This increase in activity is consistent with the improved health outcome and prolonged survival of treated mice. A representative video recording of untreated and USSC-treated C7^hypo^ mice is available in the supplemental information (Video S1).

Video S1. Representative recordings of C7hypo mouse movement with and without USSC treatment Five-minute recordings of 1-week-old untreated (left panel, red) and USSC-treated (right panel, green) C7hypo mice. Prior to recording, each mouse was habituated for 5 min in a clean cylindrical chamber.

In addition, IF analysis utilizing a human specific C7 antibody^37^^,^^38^ also revealed progressive deposition of C7 at the DEJ of USSC-treated C7^hypo^ mice (Figure S5).

To determine the role immunomodulation plays in the treatment of C7^hypo^ mice with USSCs, we measured inflammatory responses in C7^hypo^ mice with gradual disease progression from birth to adulthood. Systemic inflammation was assessed using complete blood counts (CBCs) and surrogate biomarkers, including the neutrophil/lymphocyte ratio (NLR) and monocyte/lymphocyte ratio (MLR), both of which are elevated in various malignancies, autoimmune diseases, cardiovascular conditions, and nonhealing diabetic wounds, and are associated with poor prognosis.^39^^,^^40^^,^^41^^,^^42^^,^^43^^,^^44^^,^^45^^,^^46^ Additionally, the systemic immune-inflammation index (SII) was calculated as (neutrophils × platelets)/lymphocytes, serving as a comprehensive hematological parameter reflecting immune and inflammatory states. We observed a significant overall elevation in neutrophil and monocyte counts (Figure 4D), as well as all of these biomarkers (Figure 4E), in C7^hypo^ mice when compared with WT mice. Additionally, we found that the most pronounced systemic inflammation in C7^hypo^ mice occurred within the first postnatal week (Figure S6), approximately the same time as when edema appears in these mice. C7^hypo^ mice treated weekly with USSCs had significantly lower counts of neutrophils and monocytes compared with untreated C7^hypo^ mice, and their NLRs, MLRs, and SII were comparable with WT mice (Figures 4D and 4E). Treated C7^hypo^ mice also did not exhibit severe edema, which suggests an underlying mechanism linking both of these outcomes that is immunomodulatory in nature.

In contrast to the rapid onset of systemic inflammation, localized immune cell infiltration developed more gradually with disease progression in the paw skin of C7^hypo^ mice (Figure S7). Interestingly, although macrophages were present in the dermis of C7^hypo^ paw skin from birth (Figure S7), IF staining found them to be mostly negative for the wound-healing-like phenotype marker CD206 until 2 weeks of age (Figure 4F). In contrast, USSC-treated C7^hypo^ paw skin exhibited CD206 expression starting from 1 week of age (Figure 4F). Similarly, USSC-treated C7^hypo^ paw skin exhibited elevated expression of CD163, another scavenger receptor and a marker of alternatively activated macrophages (Figure S8). Altogether, these results mirror what was observed with single USSC treatment, suggesting that weekly USSC treatment promoted tissue-repair macrophage differentiation, which correlated with improved survival and reductions in systemic inflammation in C7^hypo^ mice.

### USSC treatment reduced IL-1α/IL-1Ra ratios and attenuated NF-κB signaling in C7^hypo^ mice

**Figure 5:**
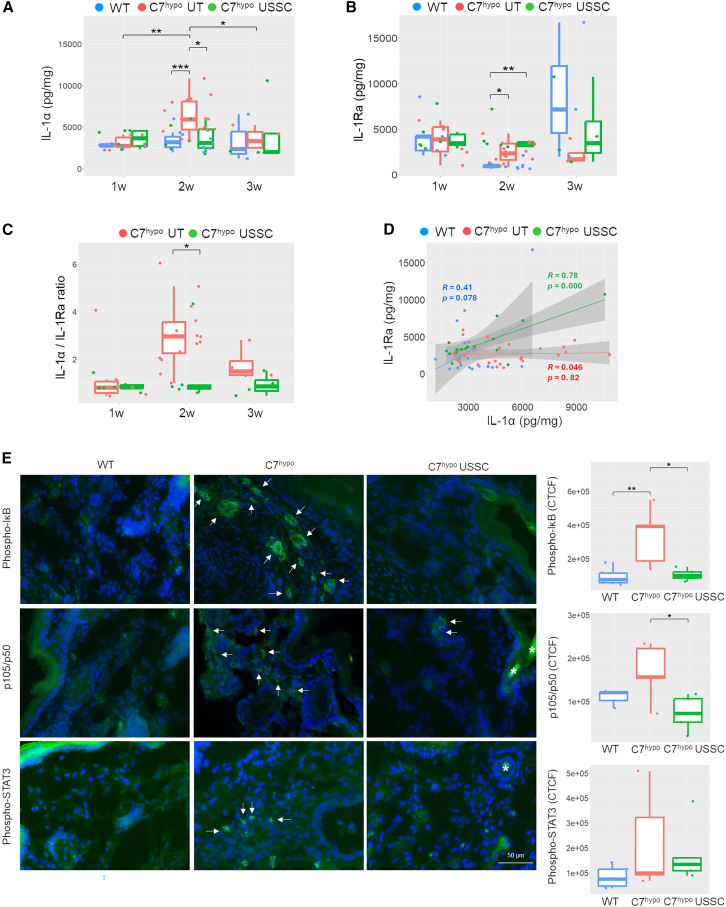
USSC treatment reduced IL-1α/IL-1Ra ratios and attenuated NF-κB signaling *in vivo* in C7hypo mice (A and B) Protein concentrations of interleukin-1 alpha (IL-1α) and interleukin-1 receptor antagonist (IL-1Ra) in paw skin lysates of WT (*n* = 22) (blue), C7^hypo^ (*n* = 39) (red), and C7^hypo^ mice treated with USSCs (*n* = 18) (green), stratified by age (1w = 1 week old, 2w = 2 weeks old, 3w = 3 weeks old). (C) Ratios of IL-1α to IL-1Ra in paw skin lysates of untreated and USSC-treated C7^hypo^ mice, stratified by age. (D) Correlation analysis between IL-1Ra and IL-1α concentrations in WT (blue), C7^hypo^ (red), and USSC-treated C7^hypo^ mice (green) paw skin lysates. Pearson correlation coefficient (*R*) and *p* value (*p*) are shown. (E) Left panels: representative IF staining for phosphorylated IκB (Phospho-IκB), p105/p50, and phosphorylated STAT3 (Phospho-STAT3) in paw skin of 1-week-old WT, untreated C7^hypo^, and USSC-treated C7^hypo^ mice. Nuclei were counterstained with DAPI (blue). White arrows indicate the positively stained cells. White asterisks denote autofluorescence from hair. Scale bar, 75 μm. Right panels: blinded quantification of average corrected total cell fluorescence (CTCF) based on five randomly selected fields per mouse (*n* = 5 per treatment group). Statistical significance was determined by one-way ANOVA with Tukey’s post hoc test or two-tailed unpaired Student’s t test as appropriate. ∗*p* < 0.05, ∗∗*p* < 0.01, ∗∗∗*p* < 0.001.

Our recent studies demonstrated that the protein level of IL-1α was significantly higher than other pro-inflammatory cytokines including IL-1β in RDEB mouse skin.^10^ Interestingly, quantitative analysis of protein lysates from paw skin revealed that USSC treatment suppressed the elevation of IL-1α in C7^hypo^ mice while maintaining the amount of IL-1Ra, a natural antagonist of IL-1, resulting in significantly reduced IL-1α/IL-1Ra ratios (*p* < 0.05) (Figures 5A–5C). Particularly noteworthy was the change in the relative balance between IL-1α and IL-1Ra. In untreated C7^hypo^ mice, the level of IL-1Ra remained constant despite elevations of IL-1α (Cor = 0.046; *p* = 0.82) (Figure 5D). In contrast, USSC-treated C7^hypo^ mouse skin exhibited a significant positive correlation between the levels of IL-1α and IL-1Ra (Cor = 0.78; *p* < 0.001) (Figure 5D). In other words, the USSC-treated mice with elevated IL-1α also exhibited increased IL-1Ra, which suppresses IL-1 activity.

To determine whether the USSC-mediated reduction in IL-1α/IL-1Ra ratios affected downstream signaling, we quantified phosphorylated IκB, p105/p50, and phosphorylated STAT3 in the dermis of paw skin from WT, untreated C7^hypo^, and USSC-treated C7^hypo^ mice at 1 week of age. Average corrected total cell fluorescence (CTCF) scores for phosphorylated IκB were significantly elevated in untreated C7^hypo^ mice compared with WT controls (Figure 5E). USSC treatment significantly reduced CTCF scores for phosphorylated IκB and p105/p50, indicating that USSCs not only suppress IL-1α expression but also attenuate downstream NF-κB signaling (Figure 5E).

In contrast, CTCF scores for phosphorylated STAT3 did not show significant differences among treatment groups (Figure 5E), suggesting that USSC-mediated effects are specific to IL-1 signaling. Supporting this interpretation, while plasma IL-6 levels, a key activator of STAT3 phosphorylation, along with other cytokines IL-10, TNF, and IL-17 were elevated in C7^hypo^ mice, in accordance with previous studies,^9^ they were not reduced by USSC treatment (Figure S9), further indicating that the attenuation of IL-1 signaling is a primary mechanism underlying the therapeutic effects of USSCs.

### USSCs secreted LIF, PGE2, and G-CSF in response to IL-1α stimulation

**Figure 6:**
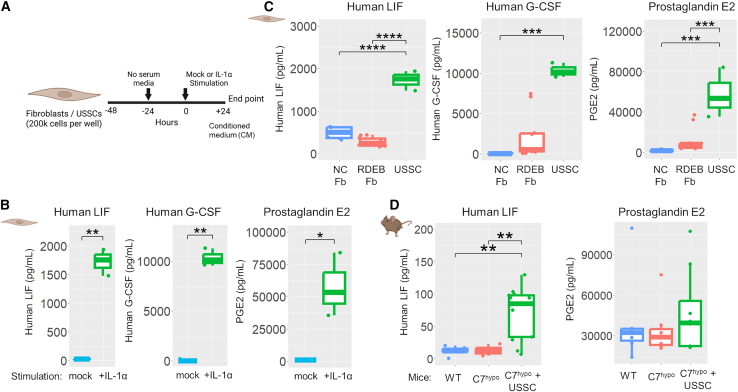
USSCs secreted high levels of immunomodulatory factors in response to IL-1α stimulation *in vitro* and inflammatory cues *in vivo* in C7^hypo^ mice (A) Schematic of the experimental timeline for IL-1α stimulation of fibroblasts and USSCs. (B) Concentrations of human LIF, G-CSF, and prostaglandin E2 (PGE2) in USSC CM 24 h after IL-1α or mock stimulation (*n* = 3 per group). Statistical significance was determined using one-tailed paired Student’s t test. (C) Human LIF, G-CSF, and PGE2 concentrations in CM from IL-1α-stimulated fibroblasts derived from normal controls (NC Fb; *n* = 6) and RDEB patients (RDEB Fb; *n* = 10), compared with USSCs (*n* = 3). Statistical significance was determined by one-way ANOVA with Tukey’s post hoc test. (D) Plasma concentrations of human LIF and PGE2 in WT (*n* = 6–12), untreated C7^hypo^ (*n* = 4–8), and C7^hypo^ mice treated with USSCs 1 day before collection (*n* = 8–13). All mice were 1–2 weeks old at the time of sample collection, with no significant differences in age between groups. Statistical significance was determined by one-way ANOVA with Tukey’s post hoc test. ∗*p* < 0.05, ∗∗*p* < 0.01, ∗∗∗*p* < 0.001, ∗∗∗∗*p* < 0.0001.

We hypothesized that USSCs were releasing factors (i.e., cytokines) in response to inflammation, specifically IL-1α, in C7^hypo^ mice that modulated their IL-1α/IL-1Ra ratios and subsequent NF-κB signaling activation. Early studies demonstrated that USSCs constitutively secrete low levels of LIF, a cytokine with pleiotropic functions in tissue regeneration and homeostasis, and produce G-CSF upon stimulation with IL-1β.^22^ G-CSF and prostaglandin E2 (PGE2) are also immunomodulatory factors known to be produced by MSCs.^47^^,^^48^ Therefore, we next measured the secretion of LIF, PGE2, and G-CSF from USSC culture *in vitro* (Figure 6A) and assessed their presence in the plasma of USSC-treated C7^hypo^ mice.

Stimulation of USSCs with IL-1α (4 ng/mL for 24 h) resulted in a significant increase in the secretion of all three factors (Figure 6B). The levels of LIF were significantly elevated compared with mock-treated control (1,726.3 ± 133.9 pg/mL vs. 25.4 ± 2.8 pg/mL; *p* < 0.01) (Figure 6B). G-CSF was undetectable in the mock control but reached 10,321.3 ± 508.9 pg/mL upon IL-1α stimulation (*p* < 0.01). Similarly, the level of PGE2 increased significantly under IL-1α-stimulated conditions compared with mock (57,617.7 ± 14,211.8 pg/mL vs. 954.1 ± 13.5 pg/mL; *p* < 0.05). In addition to IL-1α, TNF (100 ng/mL) also induced USSCs to produce LIF, but to a significantly lesser extent (Figure S10A). In contrast, IL-6 had no significant effect (Figure S10A).

Normal control and RDEB patient-derived fibroblasts were also stimulated with IL-1α in parallel to USSCs for their secretion of cytokines. Interestingly, RDEB patient-derived fibroblasts exhibited consistently lower LIF production, but higher G-CSF and PGE2 than normal controls (Figure 6C). Significantly, USSCs out-produced all three factors compared with both normal control and RDEB patient fibroblasts, which may underpin their robust therapeutic effects.

Importantly, human LIF was detected in the plasma of C7^hypo^ mice 24 h post-USSC administration (Figure 6D). Moreover, endogenous mouse LIF was also significantly increased in the paw skin of USSC-treated C7^hypo^ mice compared with WT and untreated C7^hypo^ mice at age 2 and 3 weeks (Figure S10B). To ensure that USSCs could respond to the mouse cytokine in our animal model, we also stimulated USSCs with mouse IL-1α (4 ng/mL). While the effect was not as robust as human IL-1α, mouse IL-1α was able to induce USSCs to secrete LIF (Figure S10C), confirming that the inflammatory environment from C7^hypo^ mice can trigger human USSCs to release LIF *in vivo*. These results indicate that both exogenous LIF from USSCs and endogenous LIF in treated C7^hypo^ mice are present at significant levels in the inflammatory milieu of USSC-treated C7^hypo^ mice.

As for G-CSF, despite its robust production from IL-1α-stimulated USSCs *in vitro*, it was not detected in USSC-treated C7^hypo^ plasma (data not shown). PGE2 was on average much higher in USSC-treated mice than untreated C7^hypo^ mice (47,661 ± 29,431 pg/mL vs. 34,455 ± 17,469 pg/mL); however, it was not statistically significant, due to its high plasma levels in a small subset of untreated mice (Figure 6D).

### RAW264.7 macrophages pre-conditioned with LIF expressed elevated *Il1rn* and reduced *Il1a* upon LPS stimulation

Multiple studies have suggested that MSCs possess the capability to secrete IL-1Ra on their own^49^ following stimulation with pro-inflammatory cytokines^50^ or upon co-culture with macrophages,^34^^,^^35^ which often coincided with a transition of macrophage polarization from a pro-inflammatory to a tissue-repair phenotype. However, IL-1Ra was undetectable in the USSC-conditioned medium, either under basal conditions or following stimulation with IL-1α, TNF, or IFN-γ (data not shown). Instead, in the USSC-treated C7^hypo^ mouse skin, we demonstrated that IL-1Ra predominantly originated from macrophages (CD68^+^) and neutrophils (LY6G^+^) located at the dermal-epidermal separations (Figures S11A and S11B). IL-1Ra expression within macrophages and neutrophils was also stronger in USSC-treated C7^hypo^ mouse skin when compared with untreated C7^hypo^ mouse skin (Figures S11A and S11B). The data led us to hypothesize that paracrine factors from USSCs may have modulated the immune cells, e.g., macrophages to mediate the suppression of inflammation observed with USSC treatment.

**Figure 7:**
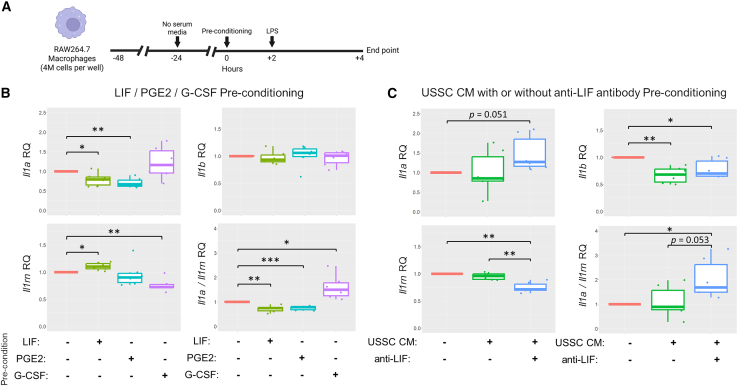
LIF suppressed IL-1α and enhanced IL-1Ra transcription in LPS-stimulated RAW264.7 macrophages (A) Schematic timeline of the experimental setup using RAW264.7 macrophages. (B and C) Relative expression (RQ) of interleukin-1α (*Il1a*), interleukin-1β (*Il1b*), interleukin-1 receptor antagonist (*Il1rn*), and the *Il1a*/*Il1rn* expression ratio following 4 h of lipopolysaccharide (LPS) stimulation, preceded by 2 h of pre-conditioning with either (B) recombinant human LIF, PGE2, or G-CSF; or (C) CM from unrestricted somatic stem cells (USSC CM), with or without neutralizing anti-human LIF antibody (anti-LIF). All experiments were conducted in three biological replicates with two technical replicates per condition. RQ values were normalized to *Gapdh* expression and compared with LPS-stimulated macrophages pre-conditioned in control medium. Statistical significance was determined by two-tailed paired Student’s t tests comparing treatment and control groups. ∗*p* < 0.05, ∗∗*p* < 0.01, ∗∗∗*p* < 0.001.

To bridge the observations that USSCs secrete LIF, G-CSF, and PGE2 in response to IL-1α stimulation and that macrophages in USSC-treated C7^hypo^ mouse skin express more IL-1Ra, we constructed *in vitro* experiments using a murine macrophage cell line (RAW264.7). Lipopolysaccharide (LPS) has been demonstrated to bind to the Toll-like receptor 4 on macrophages, leading to activation of IL-1-related genes including *Il1a*, *Il1b*, and *Il1rn*, which encode IL-1α, IL-1β, and IL-1RA, respectively.^51^ RAW264.7 macrophages were pre-conditioned with recombinant LIF, recombinant G-CSF, or PGE2 for 2 h, followed by stimulation with LPS for 4 h. Cells were then harvested for RNA extraction and analyzed by RT-qPCR to assess the expression of IL-1-related genes (Figure 7A). We noted that none of these factors had any significant effect on modulating *Il1b* expression (Figure 7B). Moreover, G-CSF pre-conditioning significantly reduced *Il1rn* expression and modestly increased *Il1a* expression, resulting in significantly elevated *Il1a*/*Il1rn* ratios. However, consistent with our hypothesis, pre-conditioning with either LIF or PGE2 significantly reduced *Il1a* expression and lowered *Il1a/Il1rn* ratios. LIF pre-conditioning also significantly elevated *Il1rn* expression (Figure 7B).

We next evaluated the effects of pre-conditioning with IL-1α-stimulated USSC-conditioned medium (USSC CM), with or without neutralizing LIF antibodies (anti-LIF), on the expression of IL-1-related genes in RAW264.7 macrophages stimulated with LPS. Surprisingly, pre-conditioning with USSC CM significantly reduced *Il1b* expression but had no statistically significant effects on *Il1a* or *Il1rn* expression (Figure 7C). Considering that neither LIF, PGE2, nor G-CSF significantly affected *Il1b* expression (Figure 7B), there are likely other factors secreted by USSCs that could downregulate *Il1b* expression. Importantly, while USSC CM alone did not seem to affect *Il1a* nor *Il1rn* expression, neutralization of LIF in USSC CM reversed its anti-inflammatory effect: *Il1a* trended higher and *Il1rn* was significantly lower, leading to a significantly elevated *Il1a*/*Il1rn* ratio (*p* < 0.05) (Figure 7C). Altogether, the data demonstrate a direct role of LIF alone in reducing *Il1a* and promoting *Il1rn* expression and suggest that multiple paracrine factors, including LIF, are involved in the immunomodulatory functions of USSCs.

## Discussion

This study highlights USSCs as a promising candidate for cellular therapy in patients with RDEB. USSC administration significantly improved the functional outcome, including survival and locomotor activities, led to deposition of new C7 at the DEJ, significantly promoted regenerative wound healing, and suppressed mutilating mitten deformities in C7^hypo^ mice. Furthermore, our investigations illuminated the impact of systemic and local inflammation in RDEB. Importantly, by comparing immune cell responses in C7^hypo^ mice with and without USSC treatment, we demonstrated that immunomodulation can rapidly ameliorate pathological progression in RDEB. Additionally, our investigations revealed a potential mechanism by which USSCs exert immunomodulation through secretion of paracrine factors such as LIF and PGE2 and subsequent reductions in IL-1α and IL-1α/IL-1Ra ratios.

Recent investigations established correlations between pro-inflammatory immune response and the development of fibrosis in RDEB patient-derived models and in C7^hypo^ mice.^8^ However, evidence linking inflammation as a direct facilitator at the early stages of the disease and following fibrosis is lacking. The absence of pSmad2/3 in the paw skin of neonatal C7^hypo^ mice with severe inflammation along with no apparent difference in pSmad2/3 between paws rapidly progressing to mutilating deformities and those with milder phenotypes, strongly suggests a weak link between TGF-β signaling and the early stages of fibrosis. This positions inflammation as the trigger for accelerated progression to fibrosis.

USSC treatment appeared to promote macrophages to express a wound-healing phenotype rather than a pro-inflammatory phenotype in C7^hypo^ mice, which likely played a significant role in their resolved inflammation and attenuated further fibrosis. Early in life, C7^hypo^ mice tended to exhibit fewer wound-healing macrophages, as measured by CD206 expression, which likely reflects a trend toward pro-inflammatory phenotype expression in macrophages promoted upon injury. The chronic wounding microenvironment of RDEB appears to impede the transition of macrophages out of the pro-inflammatory state, which may explain the rapid transition from hyperinflammation into fibrosis. USSCs seem to have facilitated macrophages to transition out of this inflammatory state, which may be due to the cytokines and factors they contribute to the local milieu.

LIF was identified as a major cytokine produced by USSCs, which by itself does not seem to directly affect macrophage polarization (data not shown). However, it did modulate the expression of IL-1-related genes, which can have higher-order effects in the dermal microenvironment that influence polarization. For example, while USSCs do not produce the macrophage polarizing cytokine IL-13 directly (data not shown), IL-13 was significantly elevated in the serum of RDEB mice treated with USSCs.^13^ Factors that are produced by USSCs can potentially stimulate other cell types that do produce IL-13, i.e., T helper cells, which result in the differentiation of macrophages into pro-wound-healing phenotypes. While these complex dynamics have yet to be elucidated, understanding them can uncover therapies that are maximally effective in modulating inflammation toward better patient outcomes.

The mechanism by which USSCs reduce IL-1α production and the IL-1α/IL-1Ra ratio in C7^hypo^ skin appears to be directly facilitated by LIF and PGE2 produced from USSCs. Our results illustrate that USSCs secrete LIF, PGE2, and G-CSF upon IL-1α stimulation, but their impact on macrophage IL-1-related gene expression varied. LIF and PGE2 alone reduced *Il1a* expression and LIF uniquely promoted *Il1rn* expression. Conversely, G-CSF seemed to have the opposite effect of LIF, reducing *Il1rn* expression and increasing *Il1a/Il1rn* ratios. Interestingly, while our *in vitro* results demonstrated that adding LIF neutralizing antibody to USSC CM reversed the expected immunomodulation, USSC CM alone did not lead to a significant reduction in *Il1a/Il1rn* ratios. We suggest that this may be due to the short half-life of LIF, and/or compromised function of LIF in the CM during the collection and freezing process. Another possible explanation involves the high concentration of G-CSF present in USSC CM, which may act in opposition to LIF. Neutralization of LIF during USSC CM pre-conditioning may have amplified the effect of G-CSF on *Il1rn* expression. This may also be relevant to our *in vivo* findings, where human LIF was detectable in the plasma of USSC-treated C7^hypo^ mice, while human G-CSF was not. It is likely that *in vivo* inflammatory activation of USSCs involves a broader array of stimuli beyond IL-1α, which was the sole inducer used in our *in vitro* experiments. Consequently, USSCs may be activated *in vivo* in a more selective manner that favors LIF over G-CSF production, contributing to reduced *Il1a/Il1rn* ratios and attenuated inflammation.

Beyond regulating IL-1 signaling, LIF also functions to modulate inflammation, promote regenerative wound healing, and regulate stem cell renewal,^52^ which also aligns with the therapeutic effects observed in USSC-treated C7^hypo^ mice. Importantly, these results should not suggest that G-CSF is detrimental; in fact, a clinical trial has demonstrated that G-CSF can promote wound healing in some RDEB patients.^53^ Ultimately, the effects of these cytokines should be interpreted in a context-dependent manner, rather than assuming them as a one-size-fits-all therapy for RDEB.

While LIF, PGE2, and G-CSF are prominent factors secreted by IL-1-stimulated USSCs, their full secretome was not assessed in this study. Additional immunomodulatory mediators are likely involved and warrant further investigation. Pre-conditioning with USSC CM significantly reduced *Il1b* expression in macrophages; however, none of the individual tested factors were able to replicate this effect. The mechanism by which these factors influence *Il1a* and *Il1rn* expression also remains unclear.

An important consideration in treating patients with RDEB is their predisposition to develop cSCCs.^3^^,^^54^ Studies examining the interactions of MSCs and tumors show that they can be both pro- and anti-tumorigenic.^55^ Furthermore, LIF has been demonstrated to promote a pro-invasive phenotype in fibroblasts that can enable cancer metastasis.^56^ While the anti-inflammatory and pro-regenerative properties of USSCs make it an attractive therapeutic for RDEB, it is vital to remain conscientious of any oncogenic effects they might have. Early in life treatment may reduce the risk of any potential pro-tumor effects and maximize the therapeutic capacities of USSCs to reduce chronic inflammation and prevent digit deformation.

In summary, our investigation has identified the role of immune dysregulation, leading to hyperinflammation, as an early event that perpetuates fibrosis throughout RDEB disease progression. While novel gene therapies, such as B-VEC, will be essential for C7 deposition and maintaining dermal structure in RDEB, addressing the underlying immune dysregulation will require combinational immunomodulatory therapies to optimize and improve the therapeutic response. The immunomodulatory effects demonstrated by USSCs in this study provide compelling support for future clinical investigations involving USSCs as a potential therapeutic approach in patients with refractory RDEB.

## Materials and methods

### Study design

The objective of this study was to determine the therapeutic effects of USSCs in a C7^hypo^ mouse model of RDEB, with a major focus on understanding how immunomodulation can modify the transition from inflammation to fibrosis. Specifically, we analyzed the immune cell responses in mice under three pathological conditions, the first with a subset of C7^hypo^ mice exhibiting paws with severe edema in their first few postnatal days, which quickly transitioned into digital mutilation fibrosis, the second with progressive fibrosis development (from newborn to 9 weeks) and the third with acute wounding. Both male and female mice were included in this study, since this phenotype is expressed similarly in both sexes. The sample size used in each experimental group was determined based on statistical power analysis to render statistical significance. The numbers of samples and replicates are indicated in respective figures and figure legends. The acquired data were included in the statistical analysis without exclusion. The choice for primary and secondary endpoints was based on animal welfare. In each experiment, mice were randomly divided into groups to receive vehicle or USSC administration. The investigator who measured the digit length, digit width, and wrist width of animals was blinded to the experimental groups.

For the experiments with the subset of C7^hypo^ mice that exhibit severe inflammation, we monitored signs of lesions and swelling in the paws of postnatal C7^hypo^ mice. Once the symptoms were observed, we measured the length and width of the mid digit and wrist width using a caliper. The mice with DL/DW ratios ≤1.5 were randomly divided into the PBS group and the group that received 1 × 10^6^ USSC intraperitoneal administration. The digit parameters were then measured by the same blinded investigator 1 week after USSC or PBS injection.

For the wound-healing analysis, 4- to 6-week-old C7^hypo^ mice and WT littermates were used for wound-healing studies. A full-thickness wound was created on the back of each of 4- to 6-week-old C7^hypo^ mice and WT using a 7 mm biopsy punch (Acuderm, FL). Twenty-four hours following induced wounding, 1.5 × 10^6^ USSCs in 100 μL PBS was injected i.d. using a 27G ½ʺ syringe (Becton Dickinson, Franklin Lakes, NJ) at about four places around the wound edges or via retroorbital vein (one experimental arm using WT mice). The mice injected with the same volume of PBS were used as negative controls. Digital photographs of wounds were taken before the treatment (D0) and daily after injection. The wound area was measured by tracing the wound margin, and quantitated using ImageJ software (NIH, Bethesda, MD). The percentage of wound area was calculated as an area of actual wound/area of original wound ×100.

For the studies starting from newborn age, C7^hypo^ mice with hemorrhagic blistering were randomly divided within 48 h of birth to receive 0.2 × 10^6^ USSCs in 20 μL PBS, or PBS alone as vehicle control via injection in the liver, since the liver is a primary site of hematopoiesis in fetal and neonatal mice and the human cells have been shown to rapidly enter the circulation after intrahepatic injection. For subsequent weekly doses, 1 × 10^6^ USSCs in 100 μL PBS or PBS alone were administered via intraperitoneal injection. For survival curve analysis, C7^hypo^ mice received USSC treatment for 3 weeks, and surviving mice were monitored for a total of 12 weeks. For experiments with endpoints at age 1, 2, or 3 weeks, samples were collected 1 week after the final dose. For experiments assessing *in vivo* cytokine production by USSCs, samples were collected 1 day after the final dose.

### Mouse models

C7^hypo^ mice were generated by breeding of the heterozygous mice, developed on a mixed C57BL/6 129sv background by replacing an 11-kb genomic fragment spanning exon 2 of *Col7a1* with a targeting construct containing phosphoglycerate kinase promoter-driven neomycin phosphotransferase (PGK-Neo) expression cassette.^32^ This genotype was confirmed by PCR. Mice were housed in a temperature- and humidity-controlled room with 12-h light/12-h dark cycles with food and water *ad libitum*.

### USSC, fibroblast, and RAW 264.7 macrophage culture

USSCs were derived from human umbilical cord blood mononuclear cells as we previously described.^16^^,^^17^ Briefly, mononuclear cells were obtained from the buffy coat interface following Ficoll-Paque PLUS (GE Healthcare PLUS, Uppsala, Sweden) gradient separation of human umbilical cord blood, and cultured in T75 tissue culture flasks in USSC initiation medium composed of 69% DMEM Low Glucose (Gibco, Auckland, New Zealand), 30% FBS (HyClone, Logan, UT), 1% penicillin/streptomycin solution, 10^-7^M of dexamethasone (Sigma, St. Louis, MO), and 2 mM ultra-glutamine (Lonza, Walkersville, MD). Half the medium was changed the next day, followed by weekly medium change until the appearance of colonies (up to 4 weeks). Cells were then expanded in the same medium without dexamethasone. USSCs were characterized based on immunophenotypes and expression of DLK1, as previously reported.^16^^,^^17^ USSCs were subsequently labeled with luciferase reporter gene, as previously reported, for bioluminescent tracking *in vivo*.^17^ USSCs at passages 4–8 were utilized in this study. RAW 264.7 macrophages and fibroblasts were expanded in DMEM medium containing 10% FBS as well as 50 μg/mL penicillin and streptomycin.

### USSC and fibroblast *in vitro* cytokine stimulation

To quantify LIF production from control and RDEB fibroblasts as well as USSCs, we seeded each cell line in BioLite 6 well multidishes (Thermo Scientific, Rochester, NY) at 200k cells per well. Cells were kept in 10% FBS DMEM medium overnight and were switched to DMEM medium alone for 24 h. Medium was replaced with DMEM medium without FBS containing either 4 ng/mL human IL-1α (BioLegend, CA), 100 ng/mL human TNF (BioLegend), 2 ng/mL human IL-6 (R&D Systems, MN), or 4 ng/mL mouse IL-1α (BioLegend) for 24 h. The supernatant of each cell line and condition was collected and analyzed for their LIF concentration by ELISA (R&D Systems). To collect USSC-stimulated supernatant for RAW 264.7 macrophage prestimulation, USSCs were expanded to confluency in T75 flasks (CellTreat, MA), placed in DMEM medium without serum overnight and then stimulated with 4 ng/mL IL-1α for 24 h. IL-1α medium was replaced with DMEM-only medium for 24 h. The supernatant of USSCs were collected and directly frozen at −20°C.

### RAW 264.7 macrophage LPS stimulation

RAW 264.7 macrophages, generously supplied by Dr. Bakshi’s laboratory, were seeded in BioLite 6-well plates (Thermo Scientific) at 4 million cells per well. Cells were kept in 10% FBS DMEM medium overnight and were switched to DMEM medium alone for 24 h. Their medium was then replaced with either DMEM only, DMEM containing 4 ng/mL LIF (R&D Systems), or supernatant from stimulated USSCs with or without neutralizing LIF antibody (R&D Systems) for 2 h. Cells were then washed with PBS and placed in DMEM containing 1× LPS (Invitrogen, CA) for 4 h. RNA was isolated from cells using the RNeasy Mini kit (QIAGEN, Germany) mixed with 20 μM Oligo(dT)12–18 Primer (Invitrogen), M-MuLV Buffer and Reverse Transcriptase (New England Biolabs, MA), RNase Inhibitor (New England Biolabs), and 10 μM dNTP Mix (Thermo Scientific) and reverse transcribed on the S1000 Thermal Cycler (Bio-Rad, CA). cDNA was mixed with Maxima SYBR Green/ROX qPCR Master Mix (Thermo Scientific) and IL-1α, IL-1β, and IL-1Rn primers (Origene, MD) and was subsequently amplified and analyzed on the 7300 Real-Time PCR System (Applied Biosystems, MA).

### IF staining and histological analyses

For the IF analysis of human C7, 5 μm cryosections were fixed in acetone/methanol (1:1) at −20°C for 10 min. A rabbit polyclonal antibody detecting human but not murine collagen VII (hLH7:2pAb^25^^,^^26^) was diluted 1:10,000 in 0.3% Triton X-100 and TBS-T and applied to the sections overnight at 4°C.^38^ After three washes with TBS-T, the slides were stained with an Alexa Fluor 488 goat anti-rabbit IgG (1:300 dilution). For the IF analysis of other antibodies, the sections were fixed in 4% paraformaldehyde and blocked with M.O.M. blocking reagent (Vector Laboratories, Burlingame, CA) (for antibodies raised in mouse) (Vector Laboratories) or CAS block (Life Technologies, MD). The slides were then incubated with respective primary antibodies, including anti-CD68 (no. 137001; BioLegend), anti-tryptase (NBP2-26444, Novus Biologicals, CO), anti-CD4 (no. 100506, BioLegend), anti-CD8 (no. NBP2-25195, Novus Biologicals), anti-Ly6G (no. 127601, BioLegend), anti-FoxP3 (no. NB100-39002, Novus Biologicals), anti-F4/80 (no. 123101, BioLegend), biotin anti-CD206 (no. 141713, BioLegend) and anti-IL-1Ra (AF771, R&D Systems), followed by corresponding secondary Alexa Fluor 488 or 546 antibodies (Invitrogen) or Cyanine3 Streptavidin (no. 405215, BioLegend) for Biotin anti-CD206. The slides were then mounted in Vectashield mounting medium containing DAPI (Vector Laboratories). Images were acquired using EVOS M5000 imaging system (ThermoFisher Scientific) using the same settings between the different groups in each set of experiments. Quantification of F4/80+ and CD206+ macrophages was performed in a blinded manner, using ≥4 randomly selected fields per mouse. CTCF for phospho-IκB, p105/p50, and phospho-STAT3 was calculated on a per-cell basis using the following formula:

CTCF=IntegratedDensity–(CellArea×MeanBackgroundFluorescence)

Integrated density, area of cell, and mean background fluorescence were measured using ImageJ. All analysis were conducted in a blinded fashion to prevent observer bias.

### Measurement of mouse movement time

To assess spontaneous locomotor activity, mice were individually placed in a cylindrical chamber (20.5 × 47 cm, diameter × height). Each mouse was allowed to habituate in the chamber for 5 min. Following habituation, behavior was recorded for an additional 5 min period using a video camera positioned above the chamber. The chamber was cleaned between each session and videos were recorded under consistent lighting and limited noise to minimize external influence.

Movement time was quantified by a blinded observer. Movement was defined as any locomotion from one point to another, excluding grooming and observational head movement. The total time spent in movement during the 5 min recording was calculated for each mouse. All assessments were performed in a randomized and blinded fashion to ensure unbiased analysis.

### Sample collection and analysis

Peripheral blood was collected in 1.5 mL EDTA tubes through cardiac puncture under terminal anesthesia and analyzed for CBC differential using VETSCAN HM5 Hematology Analyzer (Zoetis, PA). Plasma was subsequently collected after centrifugation of blood at 3,000 × *g* for 10 min. Paw skin lysate was prepared following lysis of stripped skin tissue in the presence of protease inhibitors (Cell Signaling Technology, Danvers, MA) and homogenization in gentleMACS M tubes using the gentleMACS Dissociator (Miltenyi Biotec, Somerville, MA). Protein concentrations were determined using Bio-Rad Protein Assay (Bio-Rad Laboratory, Hercules, CA). The level of IL-1α was quantitated using CBA Flex set (BD Bioscience), following manufacture’s recommendations. IL-1ra and LIF were quantitated using ELISA (R&D Systems).

### Statistics

Statistical analysis was performed using the rstatix package in R and GraphPad Prism 9 software. Kaplan-Meier analysis and log rank (Mantel-Cox) test was used to compare survival between experimental groups.^14^ Tukey multiple comparisons test was used to analyze the difference in wound healing between conditions and treatment groups. All numerical data were based on biological replicates and presented as means ± SEM or upper and lower quartiles. Statistical evaluation between two groups was assessed with unpaired or paired Student’s t tests where appropriate, whereas groups of three or more were assessed with ordinary one-way ANOVA test with Tukey’s HSD post hoc analysis. Probabilities (*p* values) of less than 0.05 were considered significant.

## Data availability

Data utilized to generate figures are included in the supplemental information (Table S1). Additional data will be made available on request.

## Acknowledgments

We acknowledge the scientific contribution from colleagues in the Pediatric Cancer Research Foundation Laboratory at NYMC. We would also like to thank Chandra Shekhar Bakshi, DVM, PhD, from the Immunology, Microbiology, and Pathology Department at NYMC for supplying us with RAW264.7 macrophages. This work was supported by 10.13039/501100001659Deutsche Forschungsgemeinschaft – SFB1160/3-256073931 (to A.N.) and 10.13039/100000902Pediatric Cancer Research Foundation (to M.S.C.). All animal studies were conducted using protocols approved by the New York Medical College Institutional Animal Care & Use Committee (IACUC).

## Author contributions

M.S.C. and Y.L. designed the study and supervised the research. M.A.-C., A.N., E.S., B.H., R.K., J.P., M.T., and Y.L. performed the experiments. M.A.C., A.N., W.L., H.Z., J.A., M.S.-C., and Y.L. analyzed the data and interpreted the results. A.N. provided study material and assisted in data analysis and interpretation. M.A.C., A.N., M.S.C., and Y.L. wrote the manuscript. All authors reviewed the results and approved the final version of the manuscript.

## Declaration of interests

The authors declare no competing interests.

## Supplemental information

Document S1. Figures S1–S11

Table S1. Source data for figures and statistical analyses Raw data were collected, organized into spreadsheets, and analyzed in R. Tabs include: CBC (complete blood count), cytokine Data, CTCF (corrected total cell fluorescence), movement data, stimulated stromal cells, USSC 1-day treatment, and RAW macrophage pre-conditioning (RAW 264.7 macrophages).

Document S2. Article plus supplemental information
