# Supplementary material for: Inflammatory modulation by cord blood stem cells prevented digit deformation in recessive dystrophic epidermolysis bullosa
Source: Mol Ther. 2025 Aug 28;33(11):5427–41. doi: 10.1016/j.ymthe.2025.08.038 (PMC12628145; doi:10.1016/j.ymthe.2025.08.038)
Supplement: Document S1. Figures S1–S11 [file mmc1.pdf]

YMTHE, Volume *33*

Supplemental Information

Inflammatory modulation by cord blood stem cells prevented digit deformation in recessive dystrophic epidermolysis bullosa

Morgan Anderson-Crannage, Alexander Nyström, Rahim Hirani, Edo Schaefer, Bruno Hochberg, Rebecca Kann, Jian Pan, Meijuan Tian, Hongwen Zhu, Wen Luo, Janet Ayello, Mitchell S. Cairo, and Yanling Liao

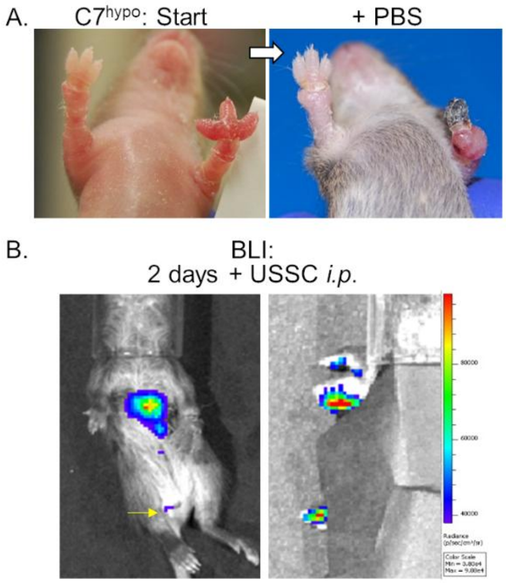

Figure S1: USSCs localized to the paw of C7^{hypo} mice after intraperitoneal injection. (A) Representative photographs of C7^{hypo} mice with edema swelling in their paws (left panel) and subsequent rapid digit necrosis 1 week later (right panel) with phosphate-buffered saline (PBS) treatment only. (B) Bioluminescence imaging (BLI) of luciferase labeled unrestricted somatic stem cells (USSCs) 2 days after intraperitoneal injection. USSC administration demonstrated a residual track of USSCs at the site of administration (a yellow arrow in the lower abdomen), accumulation in upper body (left panel), and in the paws (right panel).

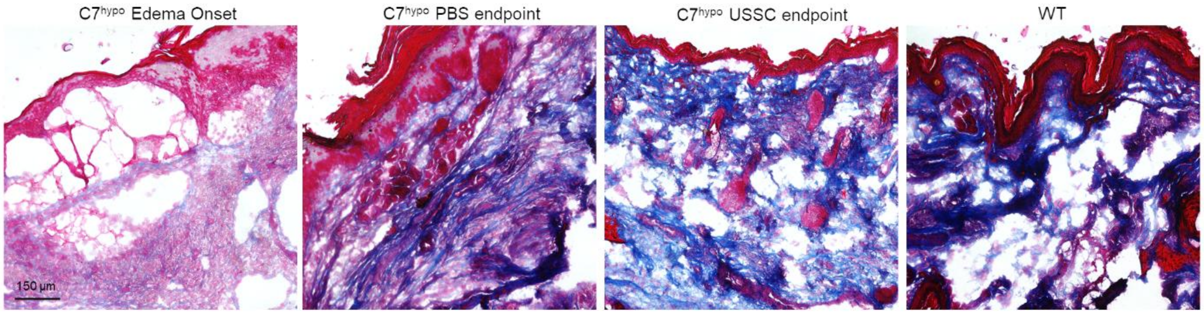

Figure S2: USSC treatment normalizes collagen organization in the dermis of C7^{hypo} mice. Masson's Trichrome staining was performed in C7hypo paw digits with edema (onset), one week post-PBS and one week post-USSC administration, along with WT control.

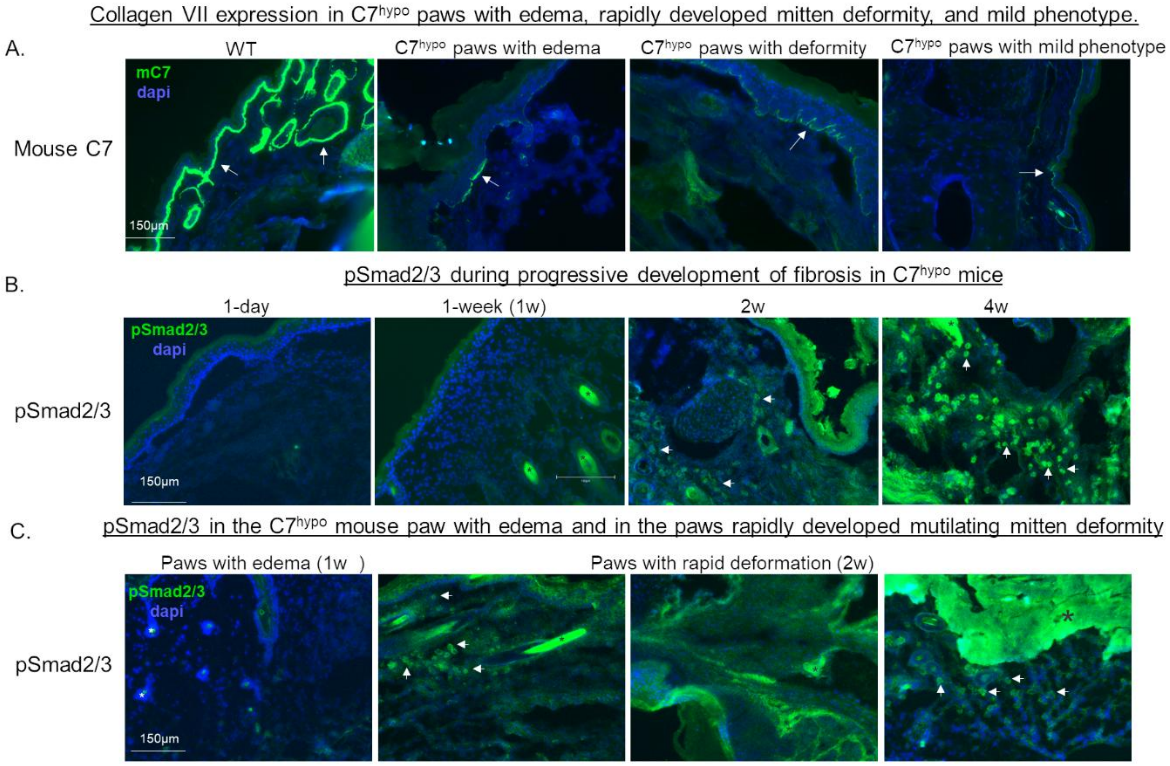

Figure S3: Comparison on C7 deposition or TGFβ signaling in the C7^{hypo} mouse skin with varied deformities. (A) Immunofluorescence (IF) staining of mouse collagen VII (C7) (green, shown by white arrows) at the dermal-epidermal junction (DEJ) in the paws of wild type (WT) and C7^{hypo} mice upon edema formation, following rapid digit deformation, and with a milder phenotype. (B-C) IF staining of phosphorylated Smad2/3 (pSmad2/3) in the paw skin of C7^{hypo} mice during (B) progressive fibrotic development at 1-day, 1-week (1w), 2-weeks (2w), and 4-weeks (4w) old and (C) rapid digit deformation at 1w and 2w old. Nuclei were counterstained with DAPI (blue). White arrows showed representative pSmad2/3 positive cells, and asterisks denote non-specific autofluorescence. Scale bar: 150 μm.

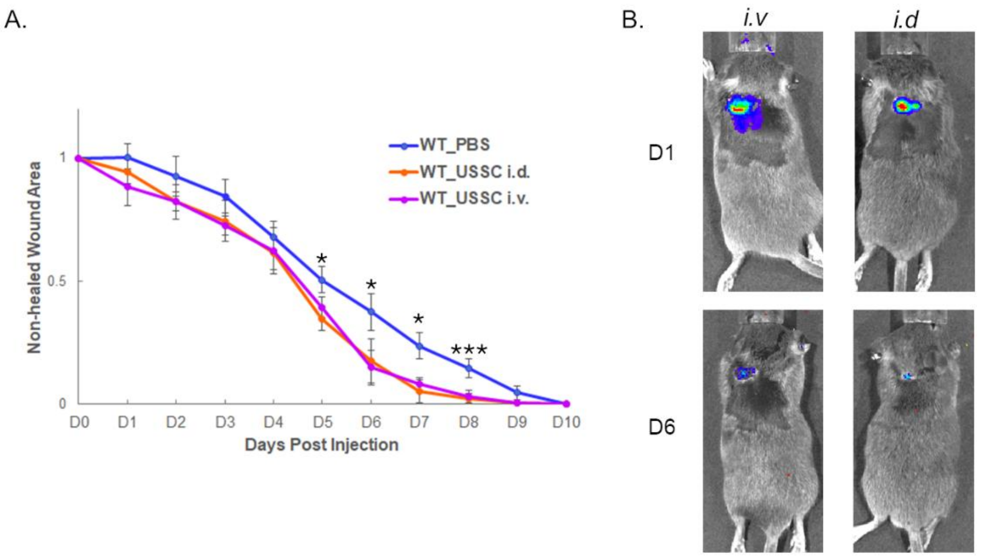

Figure S4. USSC administration enhances wound healing in WT mice through both intradermal and intravenous delivery. Wild-type (WT) mice with full-thickness excisional wounds were treated with USSCs via intradermal (i.d.) or intravenous (i.v.) injection. (A) Both delivery routes significantly accelerated wound closure compared to PBS-treated controls, with no significant difference between i.d. and i.v. administration. (B) Representative bioluminescent imaging, at D1 and D6 post USSC treatment via i.v or i.d administration.

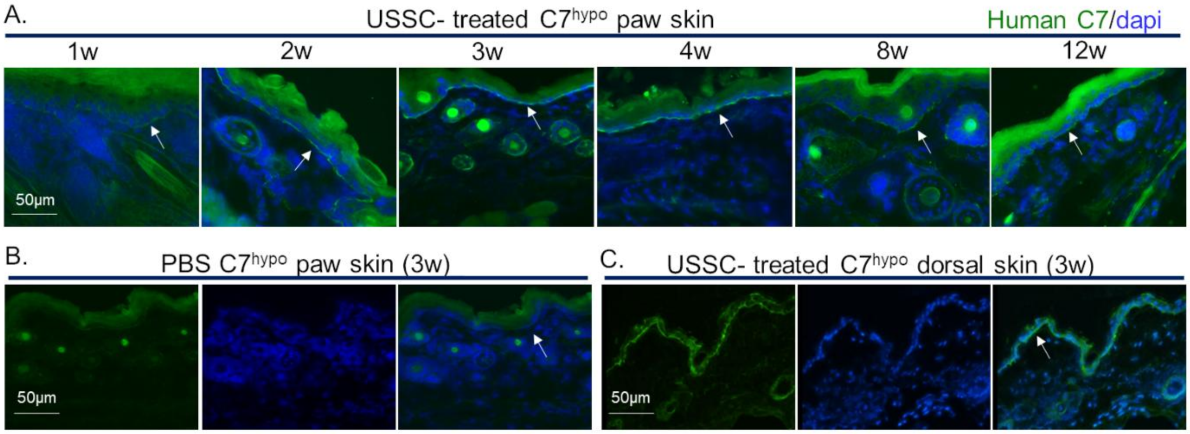

Figure S5: Weekly intraperitoneal injections of USSCs led to human C7 deposition in C7^{hypo} mouse skin. (A-D) Immunofluorescence (IF) staining of human C7 with a human-specific collagen VII pAb (green) in (A) C7^{hypo} mouse paw skin treated weekly with unrestricted somatic stem cells (USSCs) at 1-week (1w), 2-weeks (2w), 3-weeks (3w), 4-weeks (4w), 8-weeks (8w), and 12-weeks (12w) old; (B) C7^{hypo} mouse paw skin receiving weekly phosphate-buffered saline (PBS) injections at 3-weeks old; and (C) C7^{hypo} mouse dorsal skin treated weekly with USSCs at 3-weeks old. Scale bar: 50 µm. White arrows indicate the dermal-epidermal junction where C7 is localized. Nuclei were counterstained with DAPI (blue).

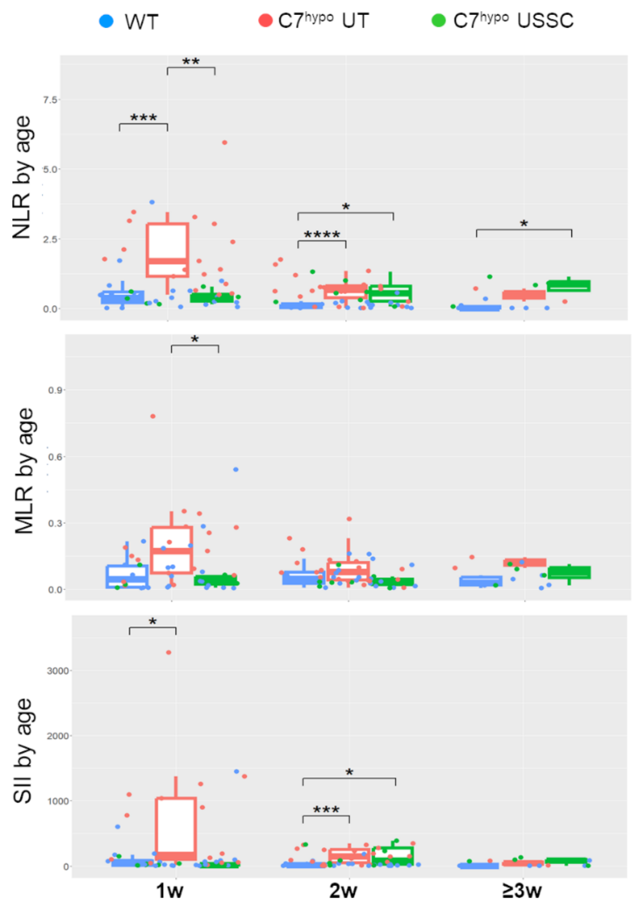

Figure S6: NLR, MLR, and SII were significantly lower in 1-week old C7^{hypo} mice treated weekly with USSCs. Neutrophil to lymphocyte (NLR), monocyte to lymphocyte ratios (MLR), and systemic immune-inflammation index (SSI) in wild type (WT) (1wk, n=23; 2wk, n=20; 3wk, n=7), C7^{hypo} untreated (C7^{hypo} PBS) (1wk, n=17; 2wk, n=20; 3wk, n=2), and C7^{hypo} mice treated with unrestricted somatic stem cells (C7^{hypo} USSC) (1wk, n=7; 2wk, n=7; 3wk, n=4) stratified by age. Statistical significances between conditions within age groups and were calculated by ANOVA with Tukey's correction. p-value < 0.05 (*), p-value < 0.01 (**), p-value < 0.001 (***), p-value < 0.0001 (****).

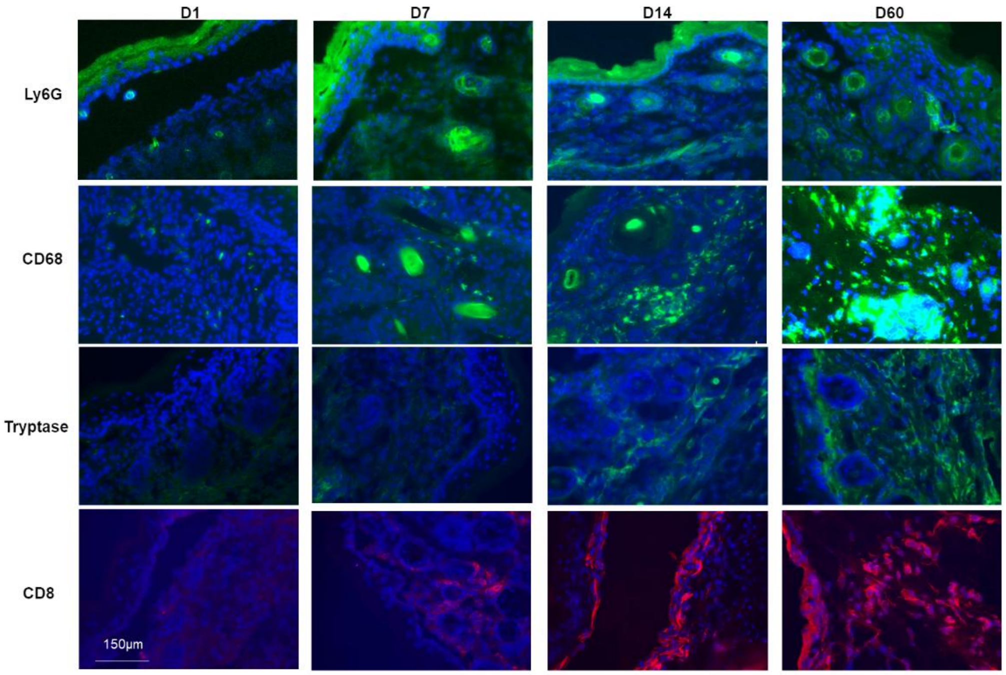

Figure S7: Immune cells progressively infiltrated the paw skin of C7^{hypo} mice.

Immunofluorescence (IF) analysis of Ly6G (green), CD68 (green), tryptase (green), and CD8 (red) on paw skin of 1-day (D1), 7-days (D7), 14-days (D14) and 60- days (D60) old C7 hypo mice. Nuclei were counterstained with DAPI (blue). Scale bar: 150 µm.

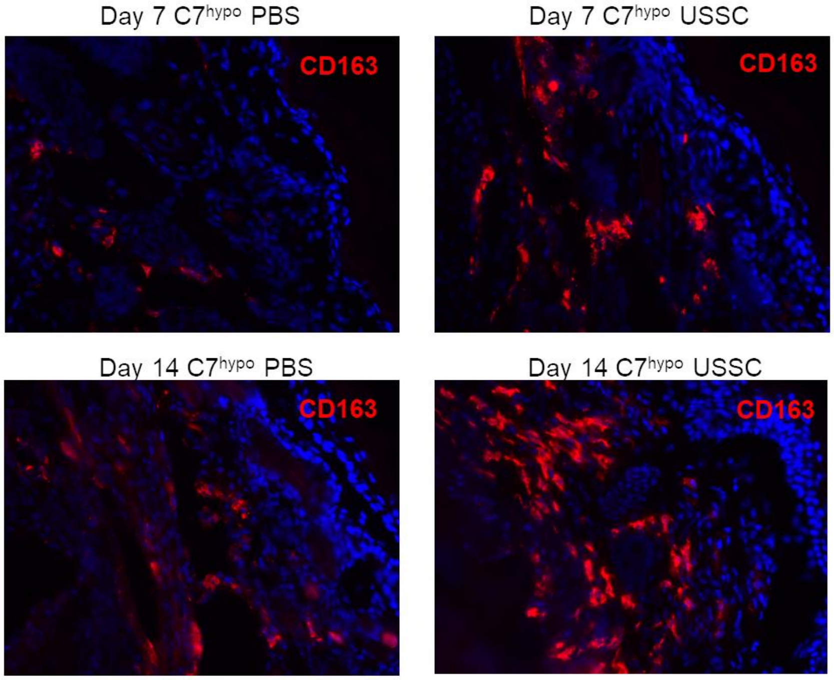

Figure S8: C7^{hypo} mice treated with USSCs exhibited more CD163+ macrophages. IF analysis of CD163 (wound healing macrophage marker) (red) in the dermis of C7^{hypo} mice treated with phosphate-buffered saline (PBS) or unrestricted somatic stem cells (USSCs) at 7-days old and 14-days old. Nuclei were counterstained with DAPI (blue).

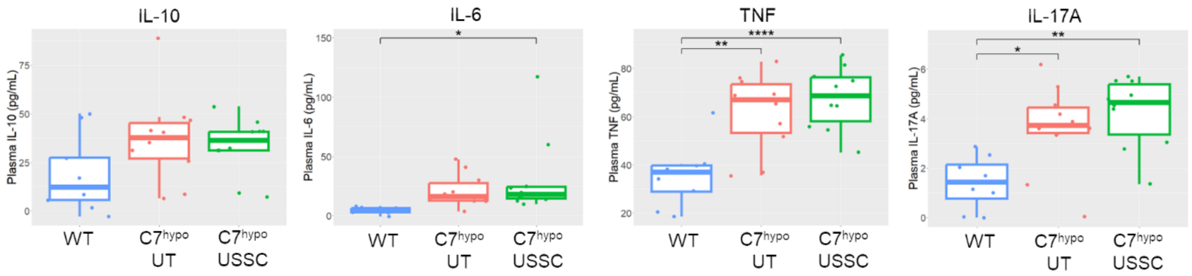

Figure S9: USSC treatment did not affect plasma concentrations of select inflammatory cytokines in C7^{hypo} mice. Plasma concentrations of interleukin-10 (IL-10), interleukin-6 (IL-6), tumor necrosis factor (TNF), and interleukin-17A (IL-17A) measured in 1-week old wild type mice (WT), C7^{hypo} PBS, and C7^{hypo}. Statistical significances between conditions (WT, C7^{hypo} UT, and C7^{hypo} USSC) were calculated by ANOVA with Tukey's correction. *p*-value < 0.05 (*), *p*-value < 0.01 (**), *p*-value < 0.0001 (****).

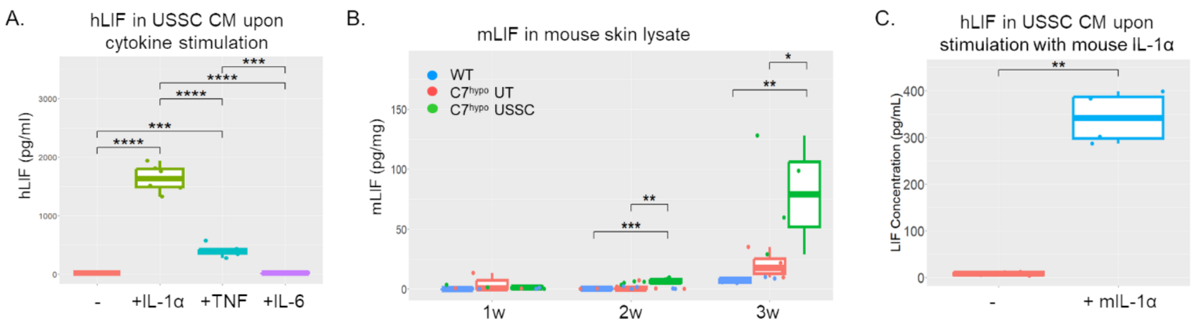

Figure S10. USSCs secreted LIF when stimulated with mouse IL-1α and elevated endogenous mouse LIF in treated C7^{hypo} mice.

(A) Concentrations of human leukemia inhibitory factor (hLIF) secreted by USSCs into conditioned media (CM) unstimulated and after stimulation with human inflammatory cytokines interleukin-1α (IL-1α), tumor necrosis factor (TNF), and interleukin-6 (IL-6). (B) Mouse LIF (mLIF) concentrations in the paw skin lysate of WT (n=13), C7hypo untreated (n=13), and C7hypo mice treated with USSCs (n=13) stratified by age (C) Human LIF concentrations secreted by USSCs into CM after stimulation with mouse IL-1α (mIL-1α). Statistical significance was determined by one-way ANOVA with Tukey's post hoc test or two-tailed paired Student's t-test as appropriate. p-value < 0.05 (*), p-value < 0.01 (**), p-value < 0.001 (***).

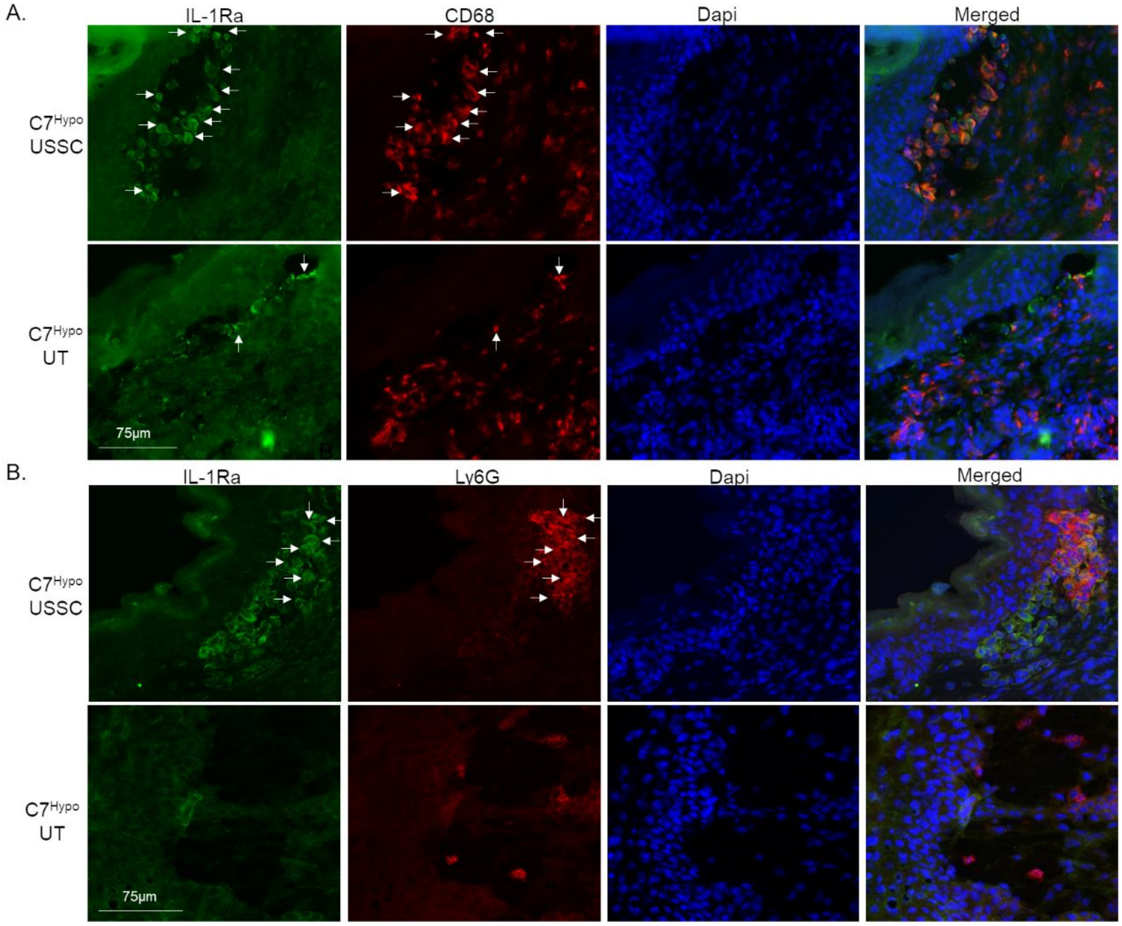

Figure S11. IL-1Ra expression in macrophages and neutrophils in USSC-treated C7^{hypo} mice.

Immunofluorescence staining for IL-1Ra (green) and (A) CD68^{+} macrophages (red) or (B) Ly6G^{+} neutrophils (red) in paw skin of 2-week-old untreated and USSC-treated C7^{hypo} mice. Nuclei were counterstained with DAPI (blue). White arrows indicate cells that are positive for both IL-1Ra and CD68 or Ly6G. Scale bar: 75 µm.

Video S1: **Representative recordings of C7hypo mouse movement with and without USSC treatment**.

Five-minute recordings of one week old untreated (left panel, red) and USSC-treated (right panel, green) C7hypo mice. Prior to recording, each mouse was habituated for 5 minutes in a clean cylindrical chamber.

Table S1. Source data for figures and statistical analyses.

Raw data were collected, organized into spreadsheets, and analyzed in R. Tabs include: CBC (complete blood count), Cytokine Data, CTCF (corrected total cell fluorescence), Movement Data, Stimulated Stromal Cells, USSC 1-Day Treatment, and RAW Macrophage Preconditioning (RAW 264.7 macrophages).
